# Neural networks engaged in tactile object manipulation: patterns of expression among healthy individuals

**DOI:** 10.1186/1744-9081-6-71

**Published:** 2010-11-24

**Authors:** Georg Kägi, John H Missimer, Eugenio Abela, Rüdiger J Seitz, Bruno J Weder

**Affiliations:** 1 Department of Neurology, Kantonsspital St. Gallen, St. Gallen, Switzerland; 2 Paul Scherrer Institute, PSI, Biomolecular Research, Villigen, Switzerland; 3 Department of Neurology, University Hospital Düsseldorf, Düsseldorf, Germany; 4 Department of Neurology, University of Bern, Bern, Switzerland

## Abstract

**Background:**

Somatosensory object discrimination has been shown to involve widespread cortical and subcortical structures in both cerebral hemispheres. In this study we aimed to identify the networks involved in tactile object manipulation by principal component analysis (PCA) of individual subjects. We expected to find more than one network.

**Methods:**

Seven healthy right-handed male volunteers (aged 22 to 44 yrs) manipulated with their right hand aluminium spheres during 5 s with a repetition frequency of 0.5-0.7 Hz. The correlation coefficients between the principal component temporal expression coefficients and the hemodynamic response modelled by SPM (ecc) determined the task-related components. To establish reproducibility within subjects and similarity of functional connectivity patterns among subjects, regional correlation coefficients (rcc) were computed between task-related component image volumes. By hierarchically categorizing, selecting and averaging the task-related component image volumes across subjects according to the rccs, mean component images (MCIs) were derived describing neural networks associated with tactile object manipulation.

**Results:**

Two independent mean component images emerged. Each included the primary sensorimotor cortex contralateral to the manipulating hand. The region extended to the premotor cortex in MCI 1, whereas it was restricted to the hand area of the primary sensorimotor cortex in MCI 2. MCI 1 showed bilateral involvement of the paralimbic anterior cingulate cortex (ACC), whereas MCI 2 implicated the midline thalamic nuclei and two areas of the rostral dorsal pons.

**Conclusions:**

Two distinct networks participate in tactile object manipulation as revealed by the intra- and interindividual comparison of individual scans. Both were employed by most subjects, suggesting that both are involved in normal somatosensory object discrimination.

## Background

A precursor of tactile exploration, tactile manipulation is a hand-object interaction in which the tight interplay between fine finger movements and kinaesthetic perception is crucial. While pure manipulation involves somatosensory control or sensory-guided movements generating independent finger movements adapted to an object, tactile exploration involves furthermore the transformation of kinaesthetic impulses into haptic information about the object being explored [1]. The essential fingers in object manipulation and exploration have been shown to be thumb and index finger [2,3].

Lesions of the primary motor, premotor and parietal cortex in humans are associated with altered patterns of tactile manipulation [4,5]. Antero-frontal lesions are related to irregular finger movements, whereas slow and irregular finger movements with increasing amplitude occur frequently in association with posterior-parietal lesions and less frequently in association with antero-parietal lesions [5].

Neurophysiological studies of specific hand movements in macaque monkeys provided additional evidence for the neural networks involved in tactile manipulation. Large distal movements as well as specific goal-directed hand movements such as grasping, holding and tearing activated neurons of the ventral premotor area (F5) as did visual presentation of 3 D objects [6]. The area F5 is directly connected with the primary motor cortex (F1) and receives rich input from the second somatosensory area (SII) and from parietal areas (PF) and AIP, the latter denoting the anterior intraparietal area located inside the intraparietal sulcus. Evidence for a similar fronto-parietal circuit in healthy humans was provided by a fMRI study which showed selective activations of the ventral premotor cortex (BA 44), the anterior intraparietal area (AIP, BA40) and of SII [7,8].

The present study investigates the tactile manipulation of spheres using event-related fMRI. As sensory-guided motor activity with little cognitive demand, the task was performed as reference in an investigation of tactile discrimination. A principal component analysis (PCA) of the reference and discrimination tasks for the group yielded a dominant component, i.e. the principal component (PC) explaining the highest proportion of variance, reflecting the concerted, directed and adaptive motion of the fingers that constitutes the basis of object manipulation and exploration [9]. The neuronal network that emerged in the component image involved the primary and secondary sensorimotor cortices, including superior parietal lobule (SPL), the dorsolateral premotor cortex and the SMA, contralateral to the active hand and the dorsal part of intraparietal sulcus on both sides. The paralimbic anterior cingulate cortex (ACC) constituted an additional node of the network. However, despite similar task performance considerable variance among subjects in the expression of the common main PC pattern was observed [9].

Variance of task-related BOLD activations among subjects is a well-known phenomenon in fMRI studies [10,11]. In addition to random sources of variation, systematic task-related variations can contribute, e.g. effects of habituation, learning, or individual subject strategies that engage different neural mechanisms of cognitive or motor processing [12-15]. Intersubject variance exceeds the relatively small variations due to cytoarchitectonic differences or spatial normalization, and is more pronounced than found in the relatively stable and reproducible activation patterns of one subject [16,17]. Indeed, recent experiments support the hypothesis that intersubject variance reflects recruitment of multiple functional networks involved in task execution [15,18]. The last reference [18] distinguishes multiple functional networks using a hybrid approach applying PCA to regions of interest derived from a categorical first-level analysis of subjects and activation conditions. A statistically more sophisticated analysis applying PCA to activation condition images also implicated multiple functional networks in a cognitive task [19].

In order to explore the intra- and intersubject variability during the manipulation of spheres repeated with a stable, periodic frequency, we have reanalyzed data investigated by Hartmann et al. [9]. The fMRI time-series image volumes of each acquisition were submitted to PCA. A similar multivariate network analysis using a modified form of principal component analysis, the Scaled Subprofile Model (SSM), was applied by Smith et al. to single-subject fMRI data to verify the neural network associated with an anatomically well-characterized simple motor task [20]. The analysis explored the spatial variability of the network among seven subjects. Our aim was to confirm the connectivity pattern observed in the group analysis, establish its reproducibility within and among subjects and explore the involvement of multiple functional networks.

PCA provides component temporal expression coefficients (eigenvariates) and image volumes (eigenimages) that can be subjected to statistical tests of task relationship and compare image volumes. The tests applied in this study consisted of computing correlation coefficients between the temporal expression coefficients and the modelled hemodynamic response of the paradigm to establish task relationship (ecc) and between component image volumes to determine topographical similarity of connectivity patterns (rcc). Additionally, PCA orders the PCs according to the degree of variance they explain in the original data set, allowing quantifying the prominence of the task-related components [21]. By hierarchically categorizing, selecting and averaging the selected task-related component image volumes across subjects according to their regional similarity, mean component images (MCIs) were derived describing prominent neural networks associated with tactile object manipulation.

We hypothesized that:

i) task-related PCs in the individual subjects are determined by the hemodynamic response model,

ii) the associated component image volumes involve the neural structures known to be involved in object manipulation and exhibited in the dominant component image of group analysis and

iii) despite similar task performance multiple functional networks emerge.

## Subjects and methods

### Subjects

Seven healthy male subjects (age range 22-44 yrs) were included. All were right-handed according to the Edinburgh handedness inventory [22,23]. None of them presented neurological or psychological disorders at the time of the study. Prior to scanning written consent was obtained from all subjects. The study was approved by the Ethics Committee of the Heinrich-Heine-University Düsseldorf in accordance with the Declaration of Human Rights in Helsinki 1975.

### Stimulation paradigm

For the present analysis, we used data from a sensorimotor task employed in a previous study as reference for somatosensory object discrimination [8]. The task consisted of sequential manipulation of nonmagnetic hard aluminium spheres with equal mass (32.5 g) and volume (11.5 cm3), which were presented to the subjects' right (dominant) hand by an investigator standing next to the scanner. Subjects were instructed to tactually manipulate the spheres in their right hand during the 5 s presentation time. No further specific instructions on hand movements were given and no explicit discrimination of the spheres was required as the objects were different neither in shape nor size. Subjects lay supine inside the scanner with their heads immobilized and their eyes closed. The investigator received the signal to present and remove the spheres via headphones which was connected to a computer outside the scanner. Each sphere was presented for 5 s while the intervals between object presentations varied pseudo randomly between 12 and 17 s implying a repetition frequency of 0.05 - 0.07 Hz. This ensured stochastic onsets of all conditions in relation to the image acquisition time, providing equal sensitivity in all slices of the acquired image volume. An fMRI scan consisted of 255*5 s event-related frames during which the subject manipulated the spheres 68 times. Each subject was scanned twice, yielding a total of 14 scans for the 7 subjects. To permit off-line analysis of the task, the sessions were recorded by a video recorder located outside of the scanner room viewing the subject close-up through a window.

### Image acquisition

Scans were acquired with a Siemens Vision 1.5 T scanner (Siemens, Erlangen, Germany) using an EPE-GE scanning sequence with TR = 5 s, TE = 66 ms, flip angle = 90°. Covering the whole brain, image volumes consisted of 30 transaxial slices parallel to the bi-commissural plane with a minimal resolution in plane of 3.125 × 3.125 mm, a slice width of 4 mm and distance between slices of 0.4 mm. In each scan 255 volumes were acquired; the first 3 were discarded from the analysis. An anatomical T1-weighted image with high resolution consisting of 128 sagittal slices and 0.9 × 0.9 mm in-plane resolution was also acquired for each subject (TR = 40 ms, TR = 5 ms, flip angle = 40°).

### Image analysis

Image pre-processing used modules of the Statistical Parametric Mapping software (SPM 2, Wellcome Department of Imaging Neuroscience, University College London, London, UK) [24] running on Matlab 6.5. Pre-processing included slice-time correction, realignment, spatial standardization to the standard brain provided by the Montreal Neurology Institute (MNI), and spatial smoothing using a Gaussian filter with an isotropic full width at half maximum of 10 × 10 × 10 mm. The dimensions of the resampled images were 79 × 95 × 68 voxels and the voxel sizes 2 × 2 × 2 mm^3^. The anatomical T1-weighted image of each subject was co-registered to the mean image of the functional images and transformed to the standard MNI space. Realignment parameters were used as confounding covariates. Data were filtered in time using a Gaussian low-pass filter of 4 s and a high-pass filter of 70 s. All data were scaled to the grand mean. The cerebral coordinates are reported in Talairach space [25]. A freely distributed Matlab script (written by M. Brett) [26] effected the transformation from MNI space.

### Principal component analysis

PCA was executed on the pre-processed fMRI time-series data using in house software written in Matlab [The Mathworks, Inc., Natick, MA] based on the algorithm described by Alexander and Moeller [27]; voxel-based PCA of single-subject fMRI data using this algorithm have been reported more recently by Andersen et al.[28] and by Smith et al. [20]. Extracerebral voxels were excluded from the analysis using a mask derived from the gray matter component yielded by segmentation of the anatomical image volume into gray matter, white matter and cerebrospinal fluid followed by the calculation of residual matrices for each of the 14 scans. From matrices whose rows corresponded to the 252 time frames of a scan and columns to the 179662 relevant voxels in a single image volume were subtracted from each element (i) the mean of voxel values of its column and (ii) the mean of voxel values of its row and (iii) added to each element the grand mean of all voxel values in the original matrices. The row, column and grand means of the resulting residual matrices vanish. Using the singular value decomposition implemented in Matlab, each residual matrix was then decomposed into 252 components. Each component consisted of an image volume i.e. eigenimage, a temporal expression coefficient i.e. eigenvariate, and an eigenvalue. The squared eigenvalue is proportional to the fraction of variance described by each component; the temporal expression coefficients describe the amount that each scan contributes to the component; and the component image displays the degree to which the voxels co-vary in the component. The temporal expression coefficients and voxel values of a principal component are orthonormal and range between -1 and 1; the orthogonality reflects the lack of statistical correlation among the principal components.

### Selection criteria for task-related principal components

Correlation between the temporal expression coefficients and the haemodynamic response (ecc) determined the task-related principal components. PCs yielding correlation coefficients exceeding 0.27 were assumed to be task-related. Based on the t-distributions governing randomly-generated correlation coefficients, this threshold reduced the probability of a false positive in the 3528 (252*14) comparisons to p < 0.05 for the 250 degrees of freedom.

To determine the prominent co-varying regions of a task-related PC, thresholds were applied to the distribution of voxel values within a PC image volume; only those voxels for which the voxel values lay in the first (negative load) or ninety-ninth (positive load) percentile of voxel values were considered. In addition, only clusters of at least 20 voxels satisfying the threshold, corresponding to one-third a resolution element, were analysed.

### Identification of similarities between PC image volumes

To establish the similarity between task-related PC image volumes, voxel correlations among the prominent co-varying regions - as defined above - of the two image volumes, were computed. Owing to the topographic pattern of the task-related PC image volumes, the voxel correlations were denoted regional correlation coefficients (rcc). Establishing a significance criterion for these coefficients required an estimate of the degrees of freedom. Since the voxels of the regions co-vary, their number is not a good estimate. The availability of a large number of principal components for each scan presumed to represent noise, as determined by, e.g. the Guttman-Kaiser criterion, suggested an empirical approach. These components described a small fraction of the variance; the total variance of all components whose eigenvalues did not satisfy the Guttman-Kaiser criterion, i.e. that the component's eigenvalue was less than the average of all eigenvalues, was typically less than 20%. For the determination of the rcc significance threshold, we assumed that the 200 of the 252 components of each scan explaining the least variance represented noise.

Taking a specific PC from each of the 14 scans, e.g. PC 8, and computing intersubject rccs with the 200 component images of the 12 scans of the other subjects yielded a distribution of 33600 rccs; this provided the basis for a least squares search to find the best fit to a theoretical t-distribution, yielding the number of degrees of freedom. For rccs determined by the 1^st ^and 99^th ^percentiles, the fit yielded 270 degrees of freedom. To determine the mean image volumes, 242 intra- and intersubject comparisons were made. In order to approach the significance threshold of p < 0.05 corrected for multiple comparisons, the theoretical distribution required the rcc to exceed 0.22. However, the computed distribution exhibited 8 outliers ranging between 0.30 and 0.33 in magnitude. This observation suggested the significance threshold of 0.30 for rccs, implying p < 0.06 corrected for 242 comparisons. In order to verify that this procedure was not overly sensitive to the percentiles chosen to define the rccs, it was repeated by correlating all non-zero voxels in the compared PC image volumes. The fit to a theoretical t-distribution yielded 1660 degrees of freedom. In order to approach the significance threshold of p < 0.05 corrected for multiple comparisons, the theoretical distribution required the correlation coefficients to exceed 0.09 in magnitude. The computed distribution exhibited 5 outliers ranging between 0.14 and 0.18. This suggested a significance threshold of 0.14, implying p < 0.04 corrected for multiple comparisons. Repeating the analysis of intra- and intersubject comparisons confirmed the evaluation of the rccs.

### Hierarchical categorization and computation of mean component images

**Figure 1:**
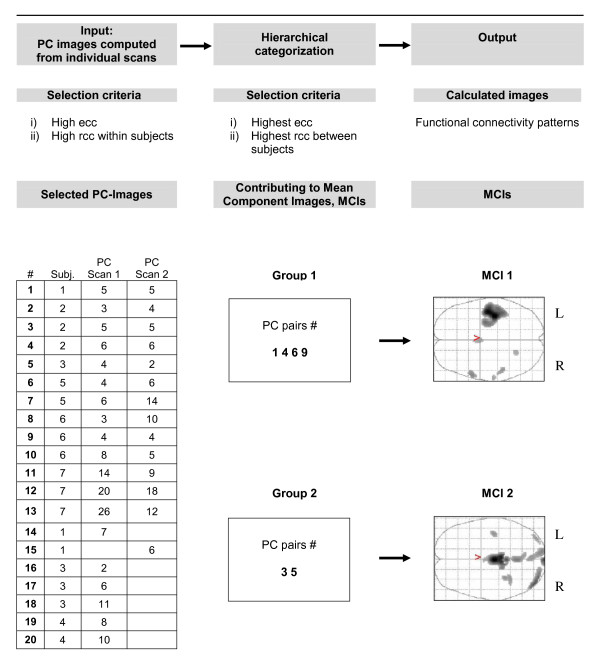
**Common neuronal networks involved in tactile object manipulation deduced from individual patterns**. Subj.: subject; PC: principal component; MCI: mean component image; ecc: temporal expression coefficient correlation; rcc: regional correlation coefficient; #: number.

In order to identify the neuronal systems represented by the task-related PCs, we established via the rccs first topographic reproducibility within subjects and second topographic similarity among subjects. In the first step, the task-related PCs in the repeated scans of each subject were matched by computing the rccs for every possible pairing. If the computation yielded more than one pairing, the most significant was accepted and the remaining pairings for that PC discarded. As shown in Figure 1 this matching procedure yielded a list of distinct paired task-related PCs for each subject; a few unmatched PCs remained.

In the second step, establishing the similarity of task-related PCs among subjects, we selected the two task-related PCs, in subjects S1 and S5, exhibiting the largest eccs (|ecc| > 0.6) and computed for each the rccs with the task-related PCs of the six remaining subjects. The rcc between these two PCs was very significant (rcc > 0.59) and showed similar patterns of rccs among the scans of the remaining subjects. The image volumes of these two PCs and of the two associated intersubject component pairs were subsumed in the first mean component image. In addition, the two patterns of rccs indicated two other subjects, S2 and S6, whose task-related PCs included component pairs or which at least one component expressed an ecc exceeding 0.55 and rcc exceeding 0.55. As indicated in Figure 1 these two pairs were also subsumed in the first mean component image, which thus comprises the average of these four pairs of task-related PC image volumes.

Computing the rccs of the first mean component image volume with all task-related PC image volumes yielded no significant correlations with two PCs of different subjects, S2 and S3, which exhibited eccs exceeding 0.44 in magnitude. The image volumes of these two PCs were significantly correlated (rcc > 0.56) and showed similar patterns of rccs among the scans of the remaining subjects. As indicated in Figure 1 the two corresponding intersubject component pairs were subsumed in the second mean component image, which thus comprises the average of two pairs of task-related PC image volumes. Computing the rccs of the second mean component image volume with all task-related PC image volumes showed that no pairs of task-related PCs with ecc exceeding 0.4 (implying p < 1.5*10^-7 ^corrected for the 14*252 two-tailed comparisons) remained that were not significantly correlated with one of the mean component image volumes 1 and 2. This indicated that no additional neuronal systems could be reliably identified.

### Conjunction images

To verify the spatial homogeneity of the task-related PCs comprising or significantly correlated with the mean component image volumes, we computed five conjunction images. To account for intersubject variability, the threshold for regional significance was relaxed to accept voxels with values lying within the 1st or 99th percentiles of the voxel value distributions. The voxel values of conjunction image volume give the number of task-related PCs for which the voxel belonged to a prominent region. For each MCI, we computed conjunction images for the task-related PCs constituting the MCI (conMCI 1, number of PCs: n = 8, conMCI 2, n = 4) and for the non-constituent task-related PCs (conMCI 1 S, n = 12, conMCI 2 S, n = 4) significantly correlated with MCI; a fifth conjunction image included those task-related PCs significantly correlated with neither MCI (conNS). Specifically 50% maps were calculated comprising the volumes delimited by the 50% isocontures, where at least 50% of the task related PCs belonged to a prominent region, i.e. overlap.

### Graphical representation of mean component images

For the purpose of additional anatomical precision, MCI images were overlaid on a surface based on representations of the MNI canonical single-subject brain using the SPM 2 surfrend toolbox (written by I. Kahn) [29] and applying appropriate voxel value thresholds such as to display voxels with values within the 1st (negative load) or 99th percentile (positive load). Surface renderings were generated for each hemisphere separately using the freely available visualization software NeuroLens (version 1.7.2) [30].

## Results

### Behavioral data

The finger movements were classified according to Roland and Mortensen into encompassing (very few), rolls (few) and dynamic digital [31]. Subjects performed mainly rolls and dynamic digital movements with thumb, index and middle finger. This movement pattern is consistent with earlier observations [3]. Thumb frequency during dynamic digital movements was on average 2.1 ± 0.2 Hz. (mean ± SD). All subjects performed equally well, and none of them lost the sphere during the task.

### Imaging data: principal component analysis

**Figure 2:**
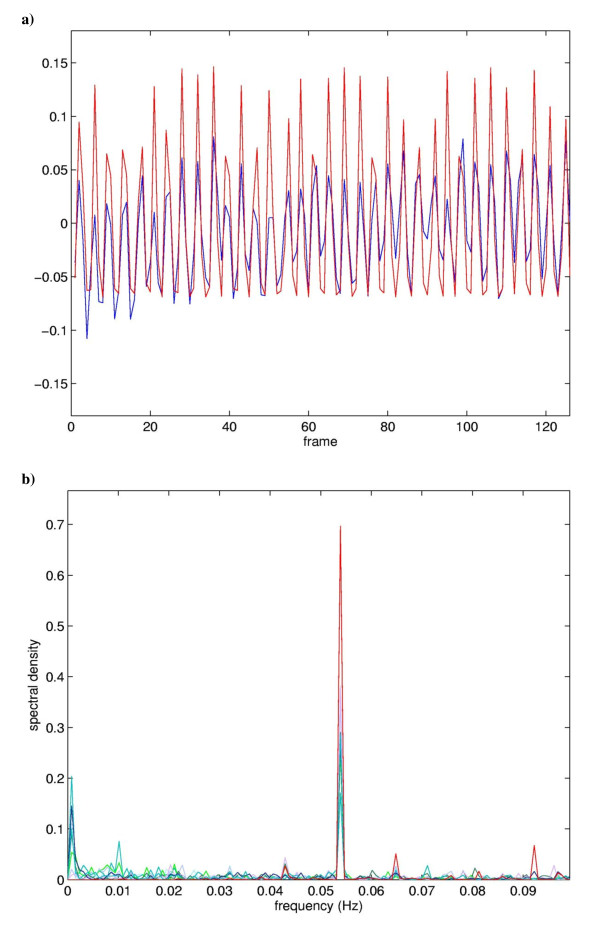
**Comparison of the mean temporal expression coefficient for MCI 1 with the modelled hemodynamic response (a) and the frequency power spectra of the underlying components temporal expression coefficients (b)**. Top: Time course of the average expression coefficient obtained from the eight PCs constituting MCI 1 (blue), superimposed on the hemodynamic model (red). The correlation coefficient between the time course and the model is 0.65. Bottom: Superposition of the frequency power spectra of the eight PCs constituting MCI 1, peak at about 0.054 Hz.

All individuals showed at least one principal component with a significant correlation (ecc > 0.27) with the hemodynamic model. A total of 33 (1.0%) of 3528 PCs in seven subjects (14 runs*252 comparisons/run) fulfilled the ecc criterion, including one marginally significant PC (see below). Furthermore, at least one significant PC emerged in each of the fourteen analyses with one exception: the PCA of the fourth subject yielded significant PCs in the first but not in the second scan. The temporal expression coefficients of all significant principal components exhibited a peak in the frequency spectrum at about 0.054 Hz, the repetition frequency of the object presentation (Figure 2a,b). All 33 task-related PCs fulfilled the Guttman-Kaiser criterion, which admitted the first 22 to 37 PCs in the 14 scans. This verifies that the choice of the last 200 PCs of each scan to represent noise in estimates of the significance threshold for rccs was a reasonable one. All but one subject evidenced significant intrasubject regional correlation coefficients (rcc > 0.3) between repeated scans indicating reproducibility; the pairs incorporated in the MCIs exhibited |rcc| > 0.69. In these subjects, the number of correlated PC pairs per subject ranged between 1 and 6 with a mean of 3.1. In one case a PC with a marginally significant ecc was included in the task-related principal components due to of a very high intrasubject rcc. (see Figure 1). The hierarchical categorization described in the methods section and in Figure 1 yielded two MCIs. As shown in Table 1 the two MCIs, derived from 12 PCs, correlated significantly with 27 of the 33 task related PCs. With one exception, the PCs not correlated significantly were marginally task related, i.e. they exhibited eccs less than 0.30. The conjunction images confirmed that the essential elements of the neural networks represented by the MCI were manifest by almost all the task-related PCs constituting the MCI as well as by all the non-constituent task-related PCs significantly correlated with MCI. In contrast, the fifth conjunction image comprising those task-related PCs that significantly correlated with neither MCI showed no distinct pattern.

**Table 1:** Regional correlation coefficients of individual PCs 1 with Mean Component Images 1 and 2.

	Scan 1^2^		MCI 1	MCI 2	Scan 2^2^		MCI 1	MCI 2
Subject	*PC*	*|ecc|*	*rcc*	*rcc*	*PC*	*|ecc|*	*rcc*	*rcc*
	
S1	5	0.62	**0.92**	-0.13	5	0.36	**0.82**	-0.20
	7	0.34	-0.10	0.53	6	0.49	-0.49	0.16
								
S2	3	0.28	-0.03	-0.31	4	0.29	0.12	0.20
	5	0.45	-0.24	**0.90**	5	0.34	-0.18	**0.84**
	6	0.46	**0.84**	-0.22	6	0.60	**-0.89**	0.20
								
S3	2	0.36	0.27	-0.36	2	0.56	-0.20	**0.94**
	4*	0.26	-0.11	**0.89**				
	6	0.28	0.28	-0.21				
	11	0.27	-0.17	-0.09				
								
S4	8	0.30	-0.39	-0.03				
	10	0.48	-0.31	0.29				
								
S5	4	0.53	**-0.72**	0.07	6	0.61	**0.84**	-0.03
	6	0.42	0.66	-0.24	14	0.33	-0.32	0.38
								
S6	3	0.33	-0.37	0.25	4	0.36	**-**0.70	0.17
	4	0.55	**-0.86**	0.36	5	0.54	**-0.80**	0.27
	8	0.32	**-**0.42	0.17	10	0.33	-0.33	-0.20
								
S7	14	0.29	-0.28	0.03	9	0.40	-0.60	0.05
	20	0.38	**-**0.35	0.03	12	0.33	-0.19	0.19
	26	0.29	0.21	0.17	18	0.40	-0.35	0.06

^1 ^33 PCs with significant temporal expression coefficient correlation (ecc) resulting from the two scans in the seven individuals are shown. ^2 ^Regional correlation coefficients (rccs) included in the computation of the mean images are in bold. PC: principal component; MCI: mean component image; *: PC with marginally significant ecc, introduced in the calculation because of the very high within subject rcc (0.78).

MCI 1 included in the 99^th ^percentile of the voxel value distribution the left primary sensorimotor cortices, including the premotor area, the paralimbic ACC on both sides and the right anterior cerebellum; the superior and inferior parietal lobe on both sides, the medial prefrontal cortex on both sides, the right posterior cingulate and the left posterior cerebellum in the 1st percentile. MCI 2 included the left primary sensorimotor cortex very circumscribed to the hand area, the precuneus on both sides, the left lingual cortex, the midline thalamic nuclei on both sides, the right posterior cerebellum and, furthermore, regions within the rostral and dorsal pons most likely representing the pontine reticular formation around the 4^th ^ventricle and the right parabrachial nucleus (lateral part) in the 99^th ^percentile; parts of the left cuneus, the lingual gyrus on both sides, the right parahippocampal gyrus, the right posterior cingulate and the right red nucleus in the 1st percentile. The regional correlation coefficient between MCI 1 and MCI 2 was not significant.

**Figure 3:**
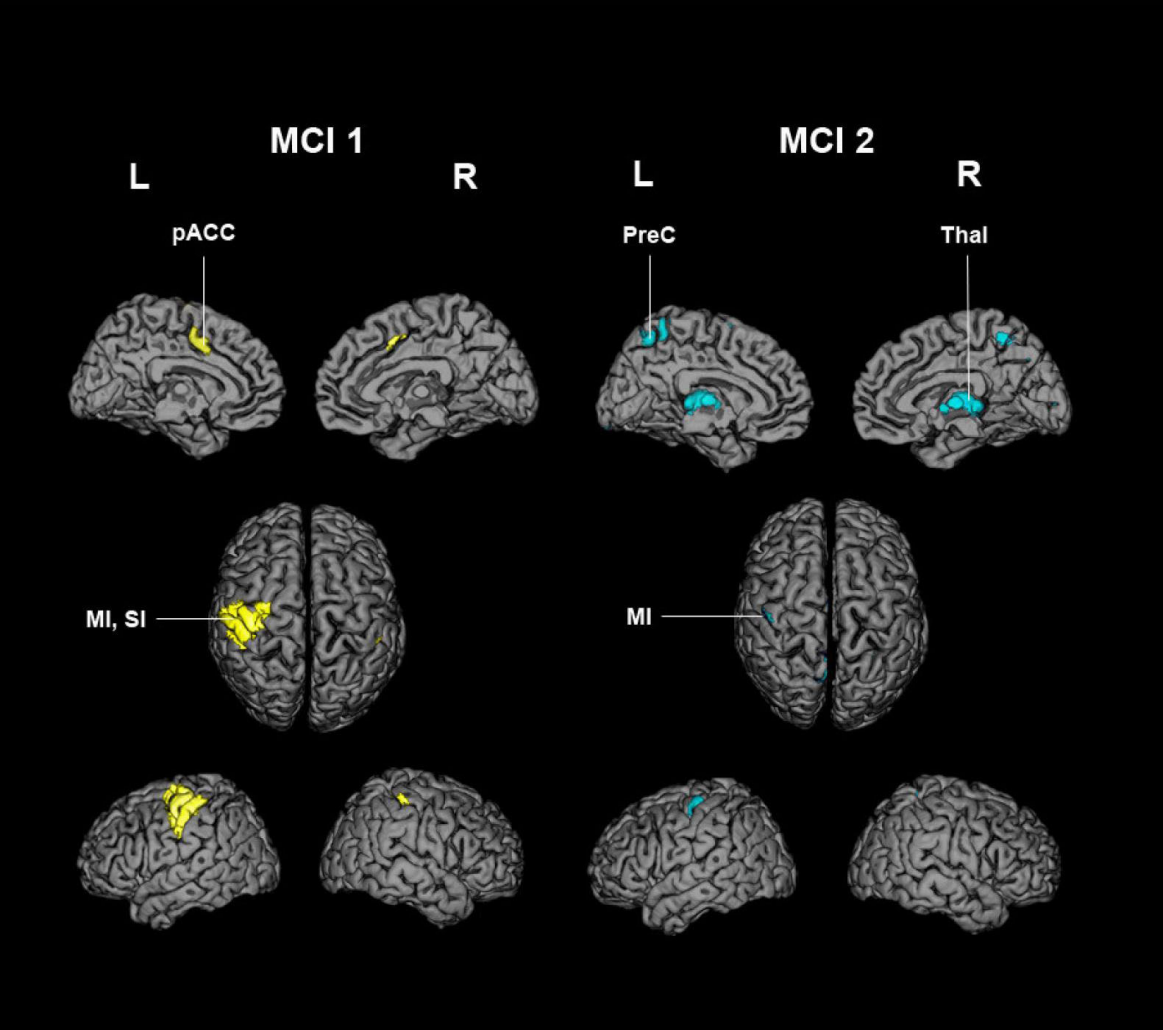
**Surface renderings of MCI 1 and 2**. Mean component images (MCI) are overlaid on a surface reconstructed canonical single-subject T1 brain in MNI space. Only voxels with values above the 99th percentile of all voxel values (positive loads) are shown. Characteristic anatomical areas of each MCI have been labelled (see text for details). L: left; R: right; MI: primary motor cortex; SI: primary somatosensory cortex; pACC: paralimbic anterior cingulate cortex (corresponding to the cingulate motor area); PreC: precuneus; Thal: thalamus

**Figure 4:**
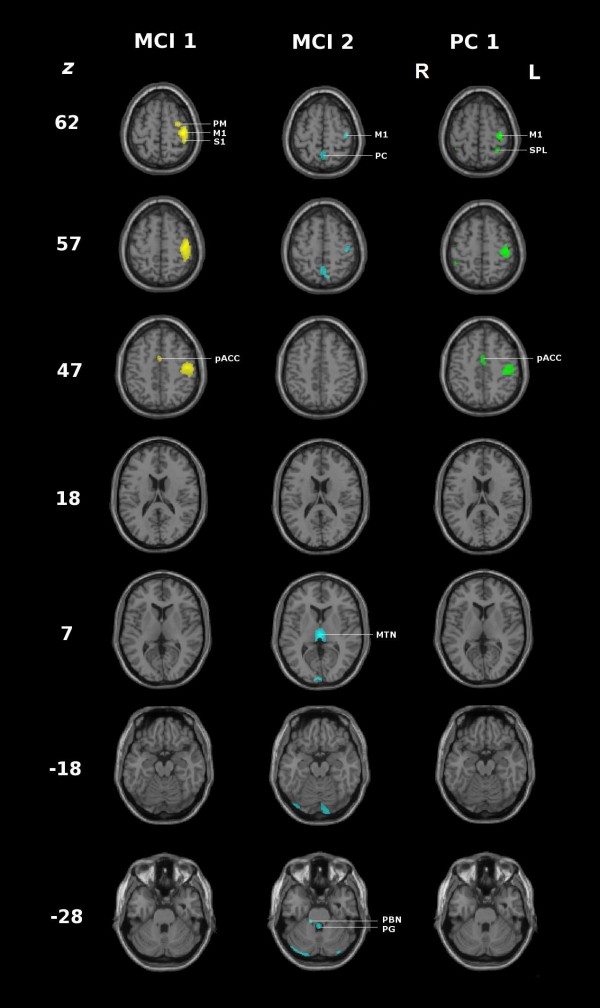
**Comparison of the positively loaded regions (99^th ^percentile) of MCI 1 and 2 and principal component 1 (PC1) of the corresponding group analysis**. Axial slices of the T1 weighted canonical single MNI subject overlaid with the positively loaded regions of the 99^th ^percentile. MCI 1 and 2: mean component image; PC1: principal component observed in the group analysis representing sensory guided motor activity [9]; z: z coordinate in Talairach space. PM: premotor cortex. MI: primary motor cortex, SI: primary somatosensory cortex, PC: precuneus, SPL: superior parietal lobule, pACC: paralimbic anterior cingulate cortex, PBN: parabrachial nucleus, PG: periventricular grey.

The description of regions in Table 2 indicates the common and specific nodes of the distributed networks represented by the two mean component images. First, both mean component images showed a significant involvement of the sensorimotor cortex contralateral to the manipulating hand (Figure 3). The contralateral sensorimotor cortex predominated in MCI 1, extending to the premotor cortex and involving the primary sensory area ipsilateral to the manipulating hand. In contrast, the implication of the sensorimotor cortex in MCI 2 was confined to the primary areas of the hand field. Second, apart from the common involvement of the sensorimotor cortex, the two mean component images exhibited considerable differences (Table 1 Figure 3 and 4), the most prominent being summarized as follows:

**Table 2:** Involved activation areas of Mean Component Images 1 and 2 with a cluster size >20 voxels.

Functional Region	Anatomical Region	x	y	z	Cluster Size	Max. Load	Conjunction Images	
**Mean Component Image 1**							**conMCI 1**	**conMCI 1S**

MI, SI and premotor c., L	Pre-and postcentral g.	-40	-20	58	1545	+0.0109	++	++
Paralimbic ACC, R/L	Medial superior frontal g.	0	3	42	61	+0.0061	++	-
SI, R	Postcentral g.	61	-21	14	22	+0.0060	++	-
Cerebellum, R	Anterior cerebellum (Lobule VI)	20	-57	-14	25	+0.0060	++	-
Temporal pole, R	Superior temporal g.	55	13	-9	78	+0.0069	+	-
Inferior parietal c., R	Inferior parietal lobule	50	-60	44	426	-0.0051	+	+
Superior parietal c., R	Superior parietal lobule	38	-59	56		-0.0034	+	-
Superior parietal c., L	Superior parietal lobule	-40	-58	53	194	-0.0039	+	-
Inferior parietal c., L	Inferior parietal lobule	-50	-58	42		-0.0038	+	-
Temporal-parieto-occipital c.	Superior temporal g.	-51	-59	29		-0.0036	+	-
Medial prefrontal c., R	Medial superior frontal g.	10	36	52	93	-0.0041	+	+
Medial prefrontal c., L	Medial superior frontal g.	-4	60	28	82	-0.0040	+	+
Precuneus, R/L	Precuneus	0	-54	38	763	-0.0050	+	+
Posterior cingulate c., R, L	Posterior cingulate g.	0	-53	23		-0.0042	+	-
Cerebellum, L	Cerebellum posterior lobe (Lobule VIIa, Crus I)	-40	-81	-25	23	-0.0037	+	-
Dorso-lateral prefrontal c., R	Middle frontal g.	46	21	39	68	-0.0037	+	+
Dorso-lateral prefrontal c., L	Middle frontal g.	-40	31	37	29	-0.0036	+	-
							
							**Additional Areas**	
							
SII, R	Postcentral g.	-60	18	16			+	-
SII, L	Postcentral g.	60	20	19			+	-
SMA, R	Medial superior frontal g.	5	4	42			+	-
CMA, L	Middle cingulate g.	0	10	35			+	-

**Mean Component Image 2**							**conMCI 2**	**conMCI 2S**

MI, SI, L	Pre- and postcentral g.	-42	-17	56	95	+0.0072	++	++
Cuneus, R, L	Cuneus	0	-94	14	69	+0.0098	++	-
Precuneus, R, L	Precuneus	0	-51	58	229	+0.0080	++	++
Lingual g., L	Lingual g.	-14	-94	-9	253	+0.0076	+	-
Thalamus, R/L	Midline thalamic nuclei	0	-21	8	607	+0.0132	++	+
Pontine reticular formation, R	Rostral pons (dorsal/medial)	14	-34	-18	56	+0.0076	-	-
Parabrachial nuclei, R/L	Rostral pons (dorsal/lateral)	0	-40	-22	89	+0.0095	++	-
Cerebellum, R	Cerebellum posterior lobe (Lobule VIIa, Crus I)	42	-80	-16	255	+0.0096	++	-
MI, R	Precentral g.	61	-8	26	87	-0.0044	+	-
Cuneus, R	Cuneus	16	-84	34	196	-0.0043	+	+
MI, L	Precentral g.	-59	-4	28	51	-0.0039	+	-
Cuneus, L	Cuneus	-12	-68	29	840	-0.0051	+	-
Lingual g., L	Lingual g.	16	-54	1		-0.0049	+	+
Lingual g., R	Lingual g.	26	-66	-3	408	-0.0048	+	+
Parahippocampal g., R	Parahippocampal g.	22	-54	-1		-0.0044	+	+
Ventral premotor c., L	Inferior frontal g.	-48	16	18	56	-0.0041	+	-
							
							**Additional Areas**	
							
Superior parietal c., L	Superior parietal lobule	-36	-42	63			+	-
Inferior parietal c., L	Inferior parietal lobule	-50	-40	54			+	-
CMA, L	Middle cingulate c.	-2	7	32			+	-

**Figure 5:**
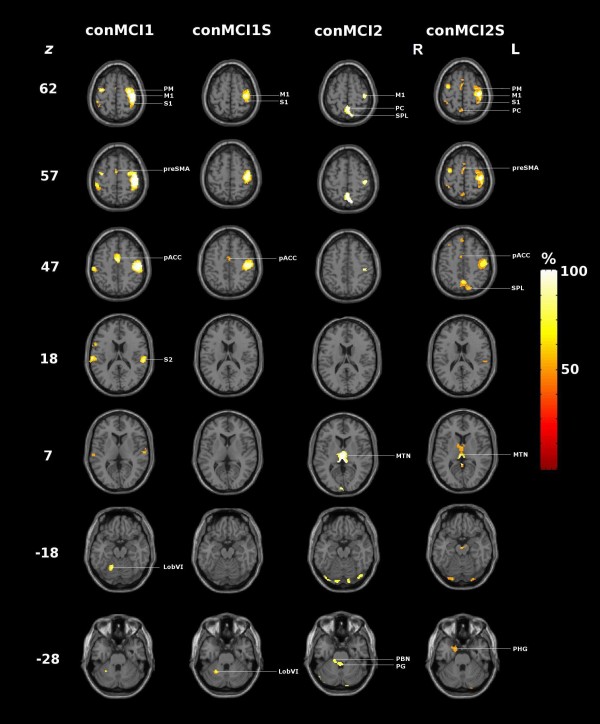
**Conjunction images of positively loaded regions (99^th ^percentile) of task-related PCs**. conMCI1, conMCI1S, conMCI2, conMCI2S : conjunction images (see text and Table 2 for details); MI: primary motor cortex, SI: primary somatosensory cortex, SII: secondary somatosensory cortex, PM: premotor cortex, PC: precuneus, SPL: superior parietal lobule, SMA: supplementary motor area, pACC: paralimbic anterior cingulate cortex, MTN: midline thalamic nuclei, PBN: parabrachial nucleus, PG: periventricular grey, LobVI: Cerebellum; Lobule VI, PHG: parahippocampal gyrus.

Functional regions are ordered according to following criteria: (1) positive loads; (2) negative loads; (3a) central -anterio-posterior cortices, i.e. primary sensorimotor areas first, followed by areas of the frontal lobes, the parietal lobes, (3b) subcortical structures and (3c) cerebellum. Indented areas belong to the preceding cluster. Coordinates (x,y,z) are given in Talairach space. The two rightmost columns represent the presence of a given brain area in the conjunction images (see Fig. 5); where '++' = 100% overlap, '+' = 50% overlap, '-' = no overlap. MCI: mean component image; conMCI: conjunction of PCS that constitute a mean component image; conMCIS: conjunction of PCs that correlate significantly with a given MCI, but were not included in its computation (see Methods for details). 'Additional Areas' lists areas present in the conjunctions, but not the MCI. pACC: paralimbic anterior cingulate cortex; CMA: cingulate motor area; MI: primary motor cortex; SI: primary somatosensory cortex; SII: secondary somatosensory cortex; SMA; supplementary motor area c.: cortex; g.: gyrus; R: right; L: left. (...) according to Schmahmann's nomenclature of the cerebellum [60]

i) MCI 1 was characterized by nodes of the paralimbic ACC on both sides and the right anterior cerebellum in the 99^th ^percentile of the voxel value distribution, and right superior and inferior parietal cortex, precuneus and posterior cingulate cortex on both sides in the 1^st ^percentile.

ii) MCI 2 was characterized by nodes of the left and right midline thalamic nuclei, pontine regions which might be localized to the pontine reticular formation and the lateral part of the parabrachial nucleus on the right side (as identified on a high-resolution computerized atlas [32]), the left and right precuneus in the 99^th ^percentile of the voxel value distribution, and the cuneus of both sides and the left lingual cortex in the 1^st ^percentile.

Of the fourteen scans in the seven subjects, the task related principal component images showed considerably varying patterns of rccs with the two mean component images (Table 1). The variations included i) significant correlation with only one MCI (8 out of 14 scans, i.e. six scans exhibiting a significant correlation only with MCI 1 and two scans only with MCI 2); and ii) significant correlations with two MCIs, i.e. MCI 1 and 2 (5 scans). In three subjects the pattern of correlations with the two mean component images was reproduced in the second scan; the remaining four subjects showed clearly different patterns in the two scans. Thus, the segregation into principal components between scans in a subject did not follow a fixed scheme but was rather characterized by fluent changes.

The mean component images showed clear correspondences to the principal component image, PC1, found in the group analysis of the same subjects during tactile manipulation and exploration [9], where PC1 was proposed to reflect the elementary processing of sensorimotor activity. MCI 1 and 2 correlated very significantly with PC1 yielding rccs, 0.76 and 0.42, respectively. Thus, the two mean component images described different patterns of neural networks mediating "sensory guided motor activity" [9].

### Conjunction images

The conjunctions of the task-related PC image volumes showed marked consistency and homogeneity across the group. As shown in Table 2 and Figure 5 the task-related PCs constituting MCI 1 and MCI 2 were characterized by extensive overlapping areas involved during tactile object manipulation. This gives direct evidence for the reproducibility of the patterns represented by MCI 1 and MCI 2. The non-constituent task-related PCs that were correlated significantly with one of the MCIs had a less distributed, but similar pattern, involving the main nodes of the respective sensorimotor network of MCI 1 and MCI 2. It should be noted that conjunction images of the constituent task-related PCs included additional positively loaded areas within the 50% threshold maps that were neither part of MCI 1 nor MCI 2. These areas include the supplementary motor area, the cingulate motor area and the posterior parietal cortex (Table 2). The task-related PCs that had no correlation to neither MCI did not also not exhibit a common neuronal network within the 50% threshold map of conNS, and were not included in the interpretation. Notably their ecc was at the limit of significance.

## Discussion

In this study we applied PCA separately to fourteen fMRI scans of seven subjects during tactile manipulation of spheres. In order to determine task-related principal components, we required significant correlations between the component temporal expression coefficients and the modelled hemodynamic response. A peak in the power spectra of the temporal expression coefficients at the repetition frequency of the task confirmed the neurobiological significance of the task-related principal components. The hemodynamic response served as sole reference; the additional reference tasks essential to conventional principal component analyses or categorical comparisons were not required. In contrast to conventional group analyses in which a principal component image volume represented the common topographic pattern of a condition to which the subjects contributed with varying component expression coefficients [9,33], the individual task-related PCs represented individual patterns describing distributed neuronal networks unique to each subject showing equal performance during tactile object manipulation.

In order to relate the task-related principal components identified in the fourteen scans, we computed intrasubject and intersubject regional correlation coefficients, measures of the topographical similarity between principal component image volumes. We confirmed the intrasubject reproducibility of the principal components and, based on discordance of the regional correlation coefficients (rccs) among subjects, found that the individual regional patterns could be expressed as two mean component images (MCI). Conjunction images served as a quality control, confirming that i) each of the task-related PCs constituting an MCI and ii) each of the non-constituent task-related PCs significantly correlated with an MCI contributed uniformly to the associated neural networks.

Our results are consistent with the view that the human brain does not function as a massive network during a specific task but rather as an assembly of smaller modular networks [34]. However, the neural networks represented by the two MCI are not closed modules but rather share common core areas of distributed neuronal networks contributing in varying degrees to the individual patterns. Subtle variations are indicated by the fact that the core areas delineated in the dominant MCI 1 appeared regularly in almost all individuals, whereas those represented in MCI 2 appeared less regularly. Furthermore, in several subjects, the same MCI contributed to more than one PC. In essence, the analysis shows that the prominent common core area represented in MCI 1 is rather stable during sensorimotor activity among repetitions and individuals but also subject to systematic variations, resulting in MCI 2. The variability is consistent with the complementary role of the different networks in a motor task. The observation of partial overlapping task-related PCs in the conjunction images may indicate additional individual variations of the patterns observed in MCI 1 and MCI 2. These areas are part of an extended sensorimotor network (Table 2).

**Figure 6:**
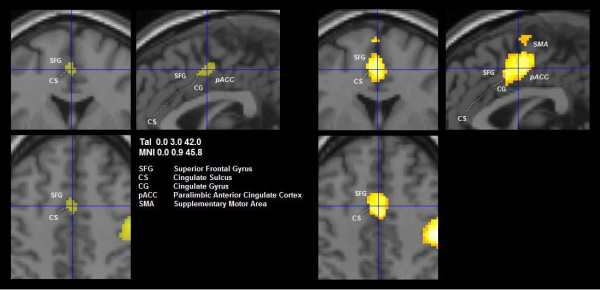
**Significant positively loaded clusters in the anterior cingulate**. Close-up views of axial (bottom left), coronal and sagittal slices (clockwise) of voxels showing positive loads in MCI 1 (left hand panel) and the corresponding conjunction image (right hand panel, activation in ≥50% of individuals). The vertical line of the cross in the sagittal slice goes through the anterior commissure, the intersection with the horizontal line lies on the cluster peak (highest positive load in MCI 1). The anterior cingulate cluster comprises both the sulcus and adjacent cingulate cortex in MCI 1, and extends to the cortex of the superior frontal gyrus in the conjunction image. Note that it remains separated from the supplementary motor area, which follows more cranially (left hand panel).

Strikingly, both MCIs involved the hand area of the primary sensorimotor cortex contralateral to the manipulating hand. The implication of the sensorimotor cortices varied in size most probably reflecting the degree of voluntary control and effort exerted during the task performance [35]. Notably, MCI 1, which correlated strongly with the main PC image of the cited group analysis of the same data, reflected a concerted, directed and adaptive motion of the fingers, the basis both for object manipulation and exploration [7]. The considerably diminished involvement of the sensorimotor cortex in MCI 2, i.e. confined to the primary sensorimotor cortex of the hand area, together with the absence of the ACC suggested less volitional effort characteristic of automatically performed movements [36]. With the exception of the primary sensorimotor cortex, the nodes represented in the two MCIs do not overlap, indicating independent distributed neural networks. The dominant MCI 1 involved the primary sensorimotor cortices contralateral to the exploring hand and the ipsilateral anterior cerebellum, the classical loop activated by phasic motor activity that has been seen regularly in conventional analyses of neuroimaging data pertaining to tactile manipulation of objects [3,37]. The involvement of the dorsal part of the premotor cortex is consistent with its participation in the fronto-parietal network regulating sensory guided movements during the manipulation of objects [5]. A special and important feature of the MCI 1 pattern was the involvement of the paralimbic anterior cingulate cortex (ACC), a node shown to play an important role in monitoring motor control, sensory perceptions, cognitive functions, and attention [35,38,39]. The paralimbic ACC includes Brodmann areas 24c and 32; it has been clearly distinguished functionally by microstimulation from the dorsally located supplementary motor area (SMA) [40]. Evidence of the functions mentioned above is provided by the neural connectivity patterns, which include projections to the motor cortex and spinal cord, afferents from the dorso-medial thalamus and reciprocal connections between the dorsal ACC and the dorso-lateral prefrontal cortex [41-46]. Paus et al. [47] established the role of the paralimbic ACC in willed control of actions and localized the distinct subregions engaged specifically in manual, verbal and oculomotor activity. According to a meta-analysis of PET activation studies, the subregion of the paralimbic ACC responsible for motor hand control is located adjacent to and on both sides of the vertical plane passing through the anterior commissure [48]. This critical compartment of the paralimbic ACC has been localized in MCI 1 as shown in Figure 6. Note that in the conjunction images the partially overlapping areas extend from there to the cingulate gyrus. Behavioral studies indicate that the paralimbic ACC is involved in the optimal performance of a task performed under neutral conditions [49], i.e. by facilitating perfect adaptation of the exploring fingers to the objects. A related lesion study of a single case revealed disruption of antiphase thumb-finger opposition required during object manipulation [50]. Since the task analyzed in the present study was executed by the subjects for the first time, the activation of the paralimbic ACC may have been especially required by the novelty of the task and the associated uncertainty of goal achievement. Tactile object manipulation does not entail conflict-induced behavioural adjustments like those required by object exploration during somatosensory discrimination [49]; the absence of a region above threshold in the dorso-lateral prefrontal cortex is consistent with this lack of cognitive demand. In summary, MCI 1 appeared to represent a state of specific activation of the motor pathways through the dense projections from the paralimbic ACC to the motor cortex with extensive activation of the sensorimotor cortices.

Prominent in MCI 2 were the midline thalamic nuclei and, most likely, the right parabrachial nucleus and the pontine reticular formation. The suggested areas of interest are the most probable structures relying on comparative internet-enabled high-resolution brain maps [32]. The parabrachial nucleus is cholinergic and influences strongly wakefulness and REM-sleep [51]. The periaqueductal/periventricular grey contains dopaminergic neurons and has extensive reciprocal connections with the sleep-wake regulatory system [52]. These areas are involved in the ascending path towards the midline/intralaminar thalamic nuclei, which are involved in regulation of cortical arousal and different aspects of awareness. It has been proposed by Van der Werf et al. [53] that the midline and intralaminar thalamic nuclei play a role in awareness, with each of the groups subserving different aspects of awareness (see below).

**Figure 7:**
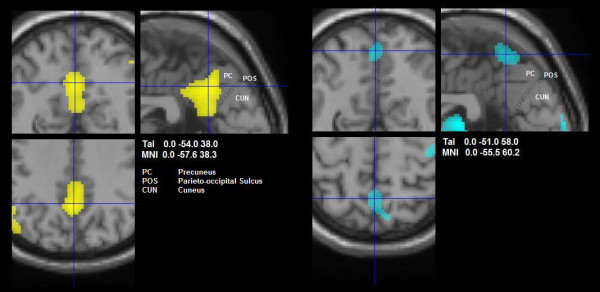
**Significant clusters in the precuneus**. Close-up views of axial (bottom left), coronal and sagittal slices (clockwise) of voxels showing negative loads in MCI 1 (yellow, left hand panel) and positive loads in MCI 2 (blue, right hand panel). In MCI 1, the cluster comprises a large area of posterior cingulate and retrosplenial cortex, commonly associated with the default mode network. In MCI 2, the cluster lies in the cranial part of the precuneus, behind the paracentral lobule. This area has recently been identified as a motor-related compartment of the precuneus (see Discussion for details).

In MCI 1 and 2 an interplay among areas involved in the baseline or resting state of brain function could be noted. The precuneus, part of the default network, yielded a negative load in MCI 1, but a positive load in MCI 2 (see Figure 7). This change took place in a compartment of the anterior precuneus that is functionally connected to the motor cortex as revealed in recent resting-state connectivity analysis in humans and monkeys [54]. We conjecture that this difference in sign may suggest less voluntary control in MCI 2 as does the lack of specific involvement of the paralimbic ACC. This subtle interplay includes the emergence of further possible constituents of the default network, i.e. the cuneus and the lingual cortex within MCI 2, which are distinguished by a negative load (except for a small positively loaded area of the right cuneus). In a recent study on resting-state functional connectivity that alternated rest with task states, both cuneus and lingual cortex have been mapped to a subcomponent of the default network that seems sensitive to previous task states [55]. Specifically, their correlation with core areas of the default network increased dynamically in a resting-state fMRI scan preceded by demanding cognitive tasks. One interpretation of their negative loading in MCI 2 might be that they indeed represent task-sensitive deactivations. However, the relationship of cuneus and lingual cortex to the default network during task states needs further investigation [56]. In summary, whereas MCI 1 seems to represent a state of cognitive motor control [35], MCI 2 suggests a state of more general alertness and automatic motor control as supported by the participation of parts of the default mode network [57].

From a physiological point of view, the fact that MCI 1 and MCI 2 appear in several subjects in the same scan implies fluctuations between specific and unspecific cortical activations due to different states of alertness. These fluctuations could be related to the two systems of subcortical-cortical projections: i.e. core cells relaying modality specific inputs in a topographically organized manner to the middle layers of specific cortical areas; ii. matrix cells receiving less-precise inputs and projecting more diffusely to layer I of the cerebral cortex [58]. They are thought to be triggered within midline thalamic nuclei, especially in the intralaminar/matrix system [51] and to reflect i) alternations between focused attention in MCI 1 with the critical involvement of the paralimbic ACC and ii) a rather broad information gathering activity in MCI 2 with tonical enhancement in the precuneus[59].

For the interpretation of the results we have to consider several limitations: First, due to the limited spatial resolution, areas of interest within the pons can only approximately be localized to a circumscribed structure. This is also true for the subregions of the midline thalamic nucleus, especially for establishing the node(s) responsible for the fluctuations between the two states of arousal suggested. Second, PCA of fMRI time-series assumes that the data can be decomposed into linearly separable spatio-temporal components. This assumption hinders the identification of probable interactions between different identified functional networks. Third, our method cannot capture the temporal relationship between two functional networks represented in MCI 1 and 2. From a theoretical point of view, one would expect some degree of temporal dissociation between them, since the two neuronal networks are spatially segregated but probably reciprocal to each other with respect to alertness. These points should be addressed by further studies.

## Conclusions

We used principal component analysis of fourteen event-related fMRI scans in seven subjects performing an elementary task, the tactile manipulation of spheres, to explore the variation in the neural patterns between repetitions within and between subjects. Correlation analysis of the principal component temporal expression coefficients and image volumes indicated that the variation could be expressed in terms of two mean component images that contributed in varying degrees to each scan, suggesting fluctuations among the dominant neural patterns. The nodes implicated by the two mean component images suggested modes of cortical activations related to different states of arousal. Moreover, the dominant mean component image volume correlated notably with the dominant component image volume of the group analysis proposed to describe sensory guided motor activity, including regions corresponding to the primary and secondary sensorimotor cortices, the superior parietal lobule (SPL), the dorsolateral premotor cortex, the SMA contralateral to the active hand, the dorsal part of intraparietal sulcus bilaterally, and the paralimbic ACC [9]. This confirmed the primacy of this neural network, whereas the remaining mean component image characterized a common individual variation of the neural networks implicated in tactile object manipulation.

## Competing interests

The authors declare that they have no competing interests.

## Authors' contributions

GK: participated in data analysis and in writing the manuscript. JHM: performed the statistical analysis and wrote parts of the paper. EA: participated in data analysis and reviewed the manuscript. RJS: participated in data analysis and reviewed the manuscript. BJW designed the study, participated in data analysis and in writing the paper and reviewed the manuscript. All authors read and approved the final manuscript.
